# An Unusual Case of Giant Bullous Disease

**DOI:** 10.1016/j.chpulm.2024.100121

**Published:** 2024-11-19

**Authors:** Kenji Yoshino, Jonathan Ioanitescu, Haiying Zhang, Tiana Endicott-Yazdani, Susan K. Mathai

**Affiliations:** a Division of Pulmonary and Critical Care Medicine, Baylor University Medical Center, Dallas, TX; b Department of Pathology, Baylor University Medical Center, Dallas, TX; c Center for Advanced Heart & Lung Disease, Baylor University Medical Center, Dallas, TX; d Texas A&M University School of Medicine, Dallas, TX

## Abstract

A 50-year-old African American woman presented to the lung transplant clinic for evaluation after experiencing gradually worsening dyspnea over the preceding 5 years. She had been diagnosed with COPD by another pulmonologist. Since her diagnosis 10 years before presentation, the patient had been on continuous supplemental oxygen therapy at 2 L/min. Her treatment regimen included a once daily combination inhaler (a corticosteroid and an ultra-long-acting ß-adrenoceptor agonist) along with an albuterol inhaler used as needed. The patient’s dyspnea limited her ability to walk half a block, and she often required a few minutes to recover after these efforts. Her symptoms were partially alleviated by use of her albuterol inhaler. In addition to dyspnea, the patient reported a nonproductive cough that was exacerbated by activity and relieved by rest. The patient’s medical history included OSA requiring positive airway pressure therapy and a hospitalization for respiratory distress due to a COVID-19 infection 12 months before presentation. She had a < 10-pack-year smoking history and childhood exposure to secondhand smoke. She had no known exposure to organic dusts or asbestos.

## Physical Examination Findings

On presentation, the patient was afebrile with a heart rate of 88 beats/min, BP of 109/76 mm Hg, and respiratory rate of 26 breaths/min. She appeared alert and without any signs of acute distress. Examination revealed the absence of breath sounds at the apices and reduced breath sounds at the bases. The cardiovascular examination revealed distant heart sounds. All other aspects of the physical examination were within normal limits, including dermatologic and musculoskeletal testing.

## Diagnostic Studies

Notable laboratory results included an elevated sedimentation rate at 40 mm/h (reference, 0-30 mm/h) and an abnormal C-reactive protein at 6.5 mg/dL (reference, 0.0-0.4 mg/dL), and mild hypercalcemia of 10.3 mg/dL (normal, 8.6-10.0 mg/dL). HIV antigen and antibody, antinuclear antibody, and antineutrophilic cytoplasmic antibody testing were negative. The patient underwent further extensive laboratory testing, revealing negative results for several other autoantibodies, including the following: anti-SSA (Ro), anti-SSB (La), RNP, Smith, Jo-1, Scl-70, anti-Jo-1, MDA-5, NXP-2, TIF1 gamma, anti-PM, Scl-100, anti-U1-RNP, anti-SS-A 52 kD IGG, and anti-SAE 1 IGG. Alpha-1 antitrypsin level was measured at 149 mg/dL (reference, 90-200 mg/dL). Aldolase was within normal range at 4.1 units/L (reference, 1.2-7.6 units/L). Interferon gamma release assay testing was negative.

**Figure 1:**
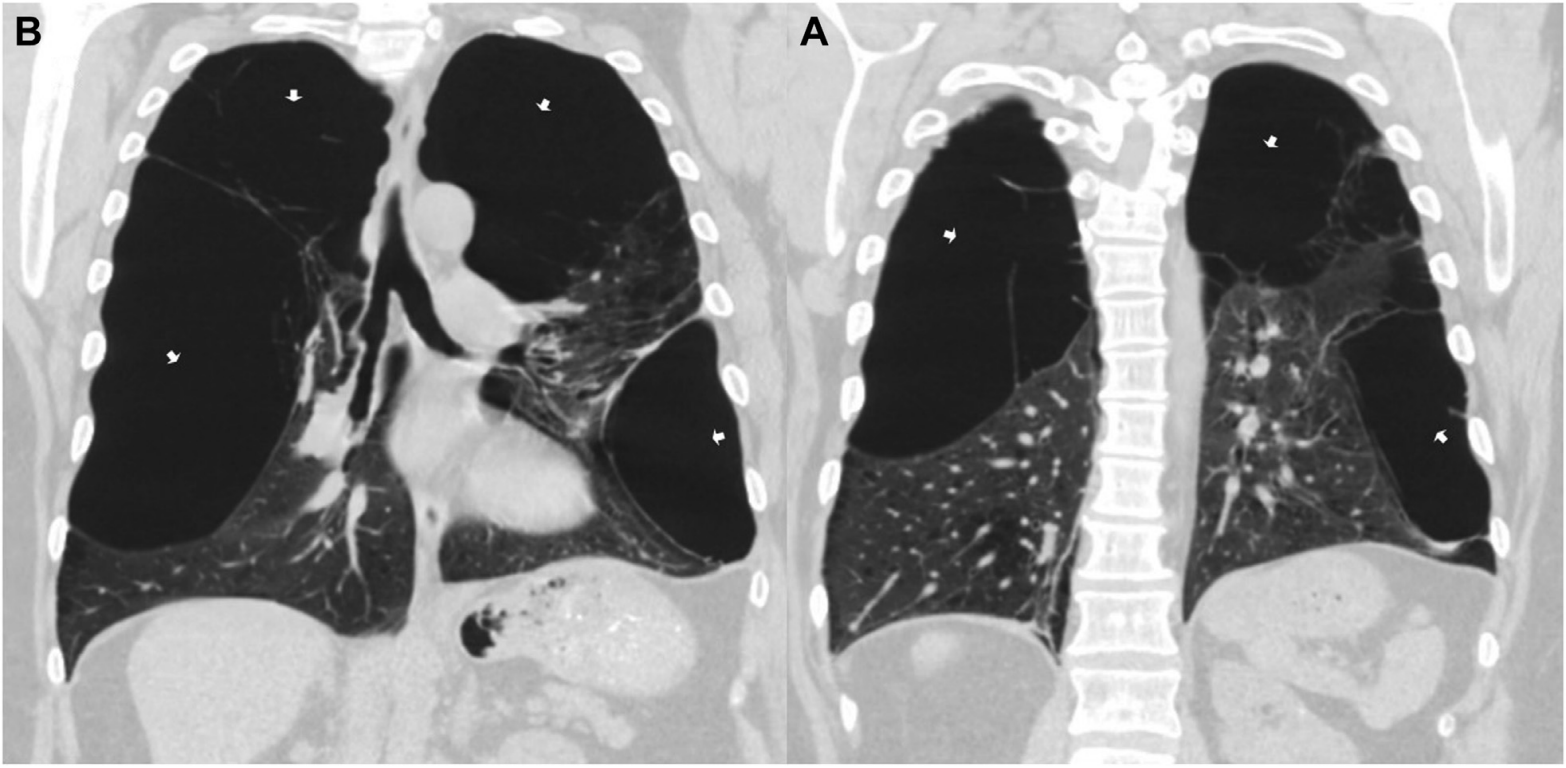
A, B, CT scan demonstrating giant bulla occupying greater than one-third of hemithorax. A, Axial slices from superior (left) to inferior (right) demonstrating bulla occupying lesions, highlighted by white arrows. B, Coronal slices from anterior (left) to posterior (right) demonstrating bulla occupying lesions, highlighted by white arrows.

The patient underwent a CT scan of the chest that revealed large bullae bilaterally (Fig 1).

Evaluation of the patient’s pulmonary function demonstrated an FEV_1_/FVC ratio of 0.59 with an FEV_1_ of 1.08 L (41% predicted) and an FVC of 1.83 L (53% predicted). Total lung capacity was measured at 5.69 L (112% predicted), and the residual volume was elevated at 3.69 L (206% predicted). These findings were consistent with obstructive physiology with prominent air trapping. A 6-minute walk test revealed that the patient required 4 L/min supplemental oxygen via nasal cannula to maintain oxygen saturations > 88%.

Given the severity of the patient’s pulmonary obstruction and the anatomic distribution of the bullae, the patient underwent lung volume reduction surgery with a video-assisted thoracoscopic surgical right upper lobe bullectomy and chemical pleurodesis.

**Figure 2:**
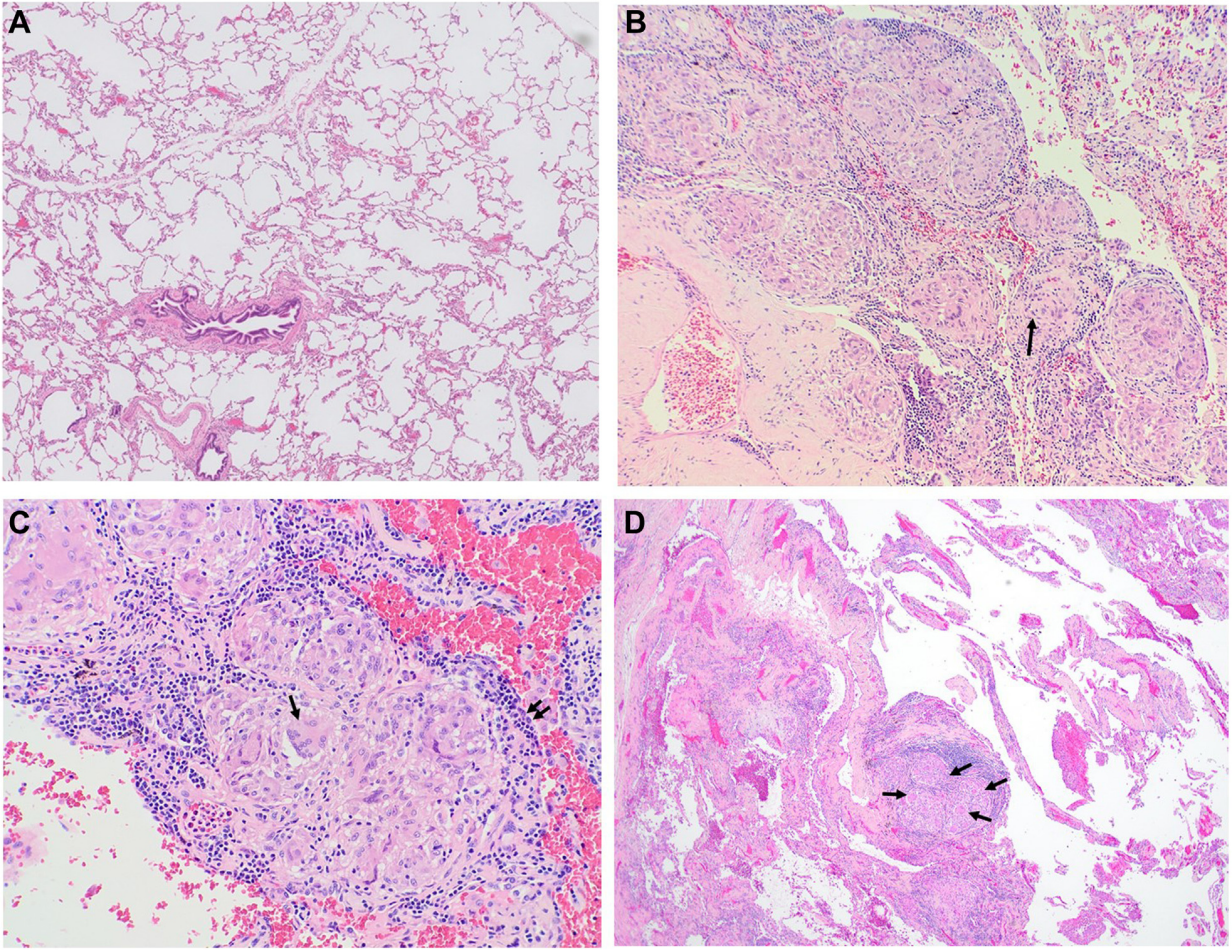
A-D, Hematoxylin and eosin staining of lung tissue. A, An example of normal alveolar tissue, magnification x40. B, Tissue from patient’s resected lung illustrating nonnecrotizing granulomas (single arrow), a cohesive aggregate of epithelioid histocytes, magnification x100. C, Tissue from the patient’s resected lung illustrating nonnecrotizing granulomas (single arrow) and multinucleated giant cell (double arrow) with a thin lymphocyte rim, magnification x200. D, Lung tissue from patient illustrating nonnecrotizing granuloma (arrows) at the site of bulla, magnification x100.

The pathology of the resected lung tissue demonstrated pleural fibrosis and well-formed nonnecrotizing granulomas involving both the lung parenchyma and pleura, without evidence of vasculitis (Fig 2). These samples were negative for fungal organisms by Grocott methenamine silver stain, negative for acid-fast bacilli by Ziehl-Neelsen stain, and negative for other bacterial organisms by gram stain.

*What is the diagnosis?*

*Diagnosis:* Bullous sarcoidosis

## Discussion

Sarcoidosis is a granulomatous disorder that can affect any organ, most commonly the lungs. More than 90% of patients with sarcoidosis have lung or intrathoracic lymph node involvement. The average age at diagnosis is between 35 and 50 years of age. Most patients with pulmonary disease present with parenchymal or airway abnormalities; giant bullous disease is a rare pulmonary manifestation of sarcoidosis, and its true incidence remains unknown.

Patients with pulmonary sarcoidosis often present with nonspecific symptoms (eg, fatigue, dry cough, exertional dyspnea). Hypoxemia is usually not seen until the advanced stages of disease. In addition to pulmonary parenchymal abnormalities, patients can develop airways inflammation and obstructive physiology and pulmonary hypertension. Skin manifestations (eg, erythema nodosum, lupus pernio) can also be present.

The most common radiologic features of pulmonary sarcoidosis include lymphadenopathy (especially hilar/mediastinal), micronodular pulmonary opacities with a peribronchiolar distribution, ground-glass opacities, and small airways disease (which can cause air trapping). Bullae as a manifestation of sarcoidosis is uncommon.

Pulmonary function testing abnormalities encountered with sarcoidosis can reveal a restrictive pattern, but obstructive physiology is also observed. Airway obstruction is thought to be caused by airway distortion due to granulomatous inflammation, lymphadenopathy causing extrinsic compression of large airways, endobronchial granulomatous inflammation, and granulomas affecting the small airways. Reduction in diffusing capacity is also possible due to granulomatous inflammation or, in some cases, due to pulmonary vascular disease.

The diagnosis of sarcoidosis is based on 3 major criteria: (1) clinical presentation, (2) the finding of nonnecrotizing granulomatous inflammation in sampled tissue, and (3) the exclusion of alternative causes for granulomatous inflammation (eg, infection, malignancy, vasculitis).

Management of sarcoidosis aims to improve lung function and quality of life through the suppression of granulomatous inflammation. Steroids are the first-line treatment for pulmonary sarcoidosis, and the dose is often determined by risk of disease progression. Recently, the SARcoidosis and CORTicosteroids (SARCORT) trial demonstrated in steroid-naïve patients with pulmonary sarcoidosis that an initial treatment regimen of 20 mg daily of prednisone was noninferior to 40 mg daily in achieving suppression of granulomatous inflammation. Doses of steroid are typically tapered slowly over 2 to 3 months after disease suppression is achieved. Additionally, because sarcoidosis is a systemic disease, patients should be closely surveilled for the development of extrapulmonary manifestations; regular eye examinations, routine electrocardiograms (EKG) and consideration of echocardiography or cardiac MRI scan, and monitoring of laboratory results to screen for hypercalcemia and renal or hepatic involvement are part of comprehensive sarcoidosis care.

For patients who are not able to taper off steroids successfully without relapse of their symptoms and/or who are grappling with glucocorticoid side effects, additional options include using steroid-sparing immunosuppressive treatments (eg, methotrexate, azathioprine, mycophenolate, leflunomide). In cases where these oral medications are not sufficiently effective, anti-tumor necrosis factor-alpha agents (infliximab, adalimumab) are considered; rituximab is also an option for refractory cases. These medications have demonstrated disease suppression efficacy in observational studies; however, robust head-to-head trials comparing efficacy of these nonsteroid options are lacking. In patients with progressive pulmonary disease despite aggressive antiinflammatory treatment, lung transplantation is considered.

Disease remission in pulmonary sarcoidosis can be seen within 2 years, and relapse after steroid discontinuation or tapering can be seen in up to one-third of patients. Relapses typically occur 1 to 12 months after steroid discontinuation or tapering.

### Clinical Course

In this case of bullous sarcoidosis, the diagnosis was established by combining clinical, radiologic, and histologic findings. Pathologically, the presence of nonnecrotizing granulomas is diagnostic when other causes of granulomatous inflammation (malignancy, vasculitis, and infection) have been excluded. In this case, lung pathology was consistent with sarcoidosis, and other potential causes of giant bullous emphysema were ruled out with laboratory testing. Clinical concern for causes of bullous disease other than smoking-related emphysema had been high given that the severity of her disease was out of proportion to the smoking history reported.

**Figure 3:**
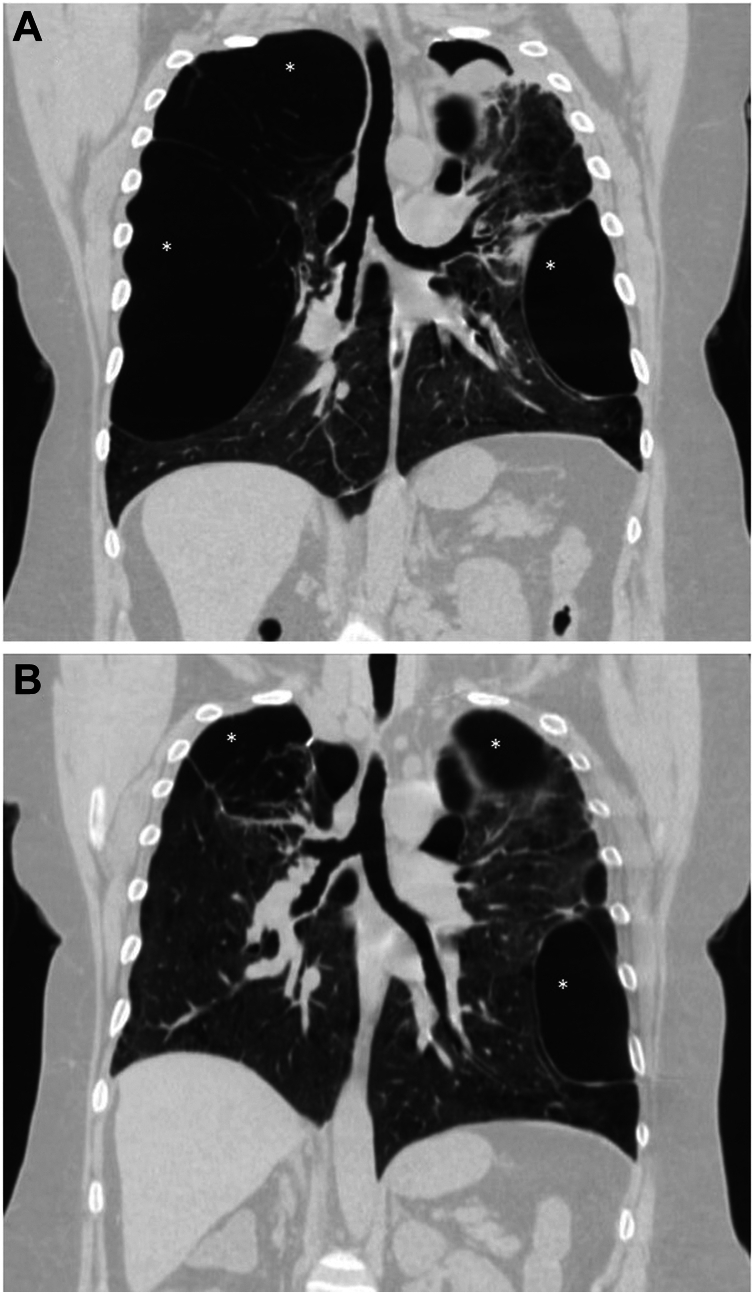
A, B, Prebullectomy and postbullectomy imaging. A, CT imaging of the patient before bullectomy, indicating extensive bullae, with the right side more affected than the left (bullae denoted by asterisks). B, Postbullectomy CT imaging, showing improvement of air trapping in the right lung, evidenced by decreased right lung volume. Bullae persist on the left side (bullae denoted by asterisks).

In this patient's case, after video-assisted thoracoscopic surgical bullectomy and immunosuppression with methotrexate and systemic steroids, there was significant improvement in the patient’s imaging (Fig 3) and pulmonary function testing. The postbullectomy pulmonary function test demonstrated improved airflows, with an FEV_1_/FVC ratio of 0.71, an FEV_1_ of 1.8 L (81% predicted), an FVC of 2.55 L (87% predicted), a total lung capacity of 5.41 L (106% predicted), and a residual volume of 2.97 L (162% predicted). Clinically, the patient’s dyspnea improved significantly; she no longer required oxygen at rest and could walk longer distances without need for supplemental oxygen.

Typically, giant bullae do not respond favorably to medical treatment alone. Lung volume reduction surgery to remove a bulla resulted in improved ventilation and lung function by reducing hyperinflation and relieving impingement of adjacent remaining functional lung tissue. In this case, surgical bullectomy played dual roles by revealing the underlying diagnosis and also providing therapeutic benefit.

## Clinical Pearls

1. *Giant bullous disease is an uncommon manifestation of pulmonary sarcoidosis.*
2. *Surgical bullectomy can potentially be diagnostic and therapeutic in cases of bullous sarcoidosis, especially if a prior tissue diagnosis of sarcoidosis has not been made.*
3. *Obstructive physiology and imaging findings out of proportion to reported tobacco smoke exposure should prompt evaluation for other causes of bullous disease.*

## Financial/Nonfinancial Disclosures

The authors have reported to *CHEST Pulmonary* the following: S. K. M. serves as an investigator for a phase 3 clinical trial in pulmonary sarcoidosis (aTyr Pharma, Inc, NCT05415137). None declared (K. Y., J. I., H. Z., T. E.-Y.).
